# Influence of Extreme Storage Conditions on Extra Virgin Olive Oil Parameters: Traceability Study

**DOI:** 10.1155/2016/7506807

**Published:** 2016-11-30

**Authors:** Alfredo Escudero, Natividad Ramos, M. Dolores La Rubia, Rafael Pacheco

**Affiliations:** ^1^Physical and Analytical Chemistry Department, University of Jaén, Campus Las Lagunillas s/n, 23071 Jaén, Spain; ^2^Chemical, Environmental and Materials Engineering Department, University of Jaén, Campus Las Lagunillas s/n, 23071 Jaén, Spain

## Abstract

This study reflects the effect of extreme storage conditions on several extra virgin olive oil (EVOO) varieties (arbequina, hojiblanca, and picual). The conditions were simulated in the laboratory, by means of heating treatments in stove at different temperatures (40 and 60°C) and times (two and three weeks). The aim is the evaluation of the deterioration of the quality parameters and minority components, which are responsible for the nutritional and therapeutic properties (fatty acids, polyphenols, pigments, and tocopherols), and organoleptic qualities. The quality criteria and limits used in this work are according to International Olive Council. The results contribute to the control of the traSceability for the commercialization of the EVOO.

## 1. Introduction

Actually, consumers and public authorities consider food safety and quality a fundamental priority because they are closely connected with the health of consumers.

Therefore, to guarantee the food safety, the policies must be based on a global and integrated approach of every link in the food chain (traceability), since all of them affect the innocuousness and the quality of the food. Likewise, the conservation and manipulation by the consumer are intimately related to the food safety, for which it is a fundamental aspect to know in which conditions the food will be altered as a consequence of the influence of different physical or chemical factors or environmental conditions.

The Regulation (EC) number 852/2004 of the European Parliament and of the Council of 29 April 2004 on the hygiene of foodstuffs [1] states the need of working under a self-checking system to ensure food safety throughout the food chain. The olive oil elaboration presents multiple risk steps that can concern its qualities and the food safety. The identification of any hazards and the application of preventive measures are crucial to obtain a safe final product with high quality. At the same time it is necessary to consider the Spanish Law 12/2013 of 2 August which gives measures to improve the functioning of the food chain [2].

The virgin olive oil is the only cooking oil that is made without the use of chemicals and industrial refining and its consumption in countries outside Mediterranean area is quickly rising. The main reasons are its sensory, antioxidant, and therapeutics properties derived from components and especially from the minor ones, as polyphenols and tocopherols, with antioxidant properties, which prevent cancer and degenerative diseases [3–6]. The health benefits of olive oil are unrivaled, and research reveals more benefits nearly every day. However, over time, oil deteriorates and its properties are altered or modified, in particular if they are used under thermal conditions which affect its stability and quality.

Every stage of the olive oil elaboration affects the organoleptic characteristics (flavor, smell, etc.) and the quality of the oil, specially of the virgin olive oil (VOO) and extra virgin olive oil (EVOO), growth, plant health, phytosanitary treatments, harvest and transport, storage, or paste preparation. During the storage and transport the VOOs and EVOOs are affected by unstable temperatures over time which alter the oil quality as a consequence of the degradation of some components.

Different papers considered the evolution of the quality parameters and the VOOs self-life during storage periods between 12 and 24 months in oil mill storage tanks [7], in bottles [8–11], in tin containers [12], in commercial conditions [13], and in home conditions [14]. The results of these studies have contributed to a better understanding of the chemical oxidation process, but they were inconclusive.

In the last decade the VOO quality prediction and the evolution of the antioxidant components during storage are themes of great interest. Several authors have analyzed the alteration of the fatty acid composition, minor components, and antioxidant activity during storage [5, 15–17]. The latter authors reported the evolution of major and minor components and oxidation indices of seven VOO samples, which differed in their initial contents of natural antioxidants significantly, during 21 months of storage at room and in darkness. The correlation between the initial composition and time to reach the upper limits established by EU Regulation is also reported.

Morelló et al. [18] observed a great decrease in the minor components in particular for the phenolic fraction and pigments while the oleic acid percentage increases in commercial arbequina VOO after 12 months at room temperature. Also, the influence of the VOO elaboration methods on the quality parameters during 18 months of storage has been studied [19]. Other research studies evaluate the oxidative capacity of the VOO under high temperature accelerated storage conditions (50°C during 8 months) [20].

In recent years, several authors [21–23] have reported results of different olive oil varieties under storage conditions between +4°C and −20°C during 12 months. The quality of the VOO remains better at low temperatures, the phenolic content decreased slightly, and the volatile compounds were unaltered. Low temperatures preserve the organoleptic properties of a fresh oil better than room temperatures.

Mulinacci et al. [24] compared the effect of a long storage period on EVOO at −23°C with the same specimens at room temperature in dark. The evolution of the phenolic composition and aromatic profile were monitored during 18 months. Increments of the phenolic composition were observed for oils stored at room temperature starting from 3 months of storage, and the frozen oils showed negligible differences in aromatic profile until 12 months of storage.

The influence of storage conditions and packing materials (clear PET, can, glass, and tetrabrick) on olive oil quality has been considered in different papers [25–30].

The tetrabrick packing preserves the original olive oil quality better than PET and glass which were responsible for a decrease of the quality parameters and minor components (pigments and tocopherols). This reveals the influence of the packing materials on the protection and quality of the olive oil.

Finally, the influence of the temperature on the vegetable oils during thermal treatment (simulating cooking) and its influence on the physical-chemical parameters have been also studied [31–33]. The studied oils were VOO, EVOO, sunflower oil (refined and high oleic content), peanut oil, and cottonseed oil and a microwave oven was used as energy source. A conventional oven was also used by some authors in order to compare results [34–37].

Therefore, as there has been little knowledge about the influence of high temperatures on the quality parameters and minor components of the EVOOs, in this research the long term storage at high temperatures is simulated. The objective of this study is to simulate inappropriate transport and storage conditions which affect the EVOO quality and could alter its “extra” denomination and evaluate the physical-chemical and sensorial properties of the EVOO under these conditions.

For these purposes, four extra virgin olive oils (picual (two brands), arbequina, and hojiblanca) from Jaén (Spain) were investigated. The accelerated assays were carried out in a laboratory oven at high temperatures and time simulating several transport or storage conditions in Andalusia region or areas with similar climatology where the temperatures in summer seasons are high.

In addition, the interest of this paper lies in the fact that the VOO export is increasing strongly and the influence of the long term transport and storage conditions has not been studied. The results will improve international transport conditions in terms of length, in order to guarantee the original characteristics in destination and to comply with the Food Safety Regulations.

## 2. Materials and Methods

### 2.1. Preparation of the Samples

Commercial samples of EVOO of the varieties: picual (two brands A and B), arbequina, and hojiblanca of the crop, in season 2012-2013 were collected from Andalusia. All the samples, bottled in clear PET, were purchased from local stores and oil mills. The olive oil samples were stored correctly up to the beginning of the experimental procedure.

Four studies were carried out: storage in a Mecánicas Científicas S.A. oven with air flow from the external environment at 40°C during two and three weeks (treatment T1 and treatment T2) and 60°C during two and three weeks (treatment T3 and treatment T4). After each treatment the samples were coded, placed in amber glass, and frozen until analysis. All the measurements were made in triplicate.

### 2.2. Quality Parameters

The quality parameters were determined according to the analytical methods described in the COI/T.15/NC number 3/Rev. 7 May of 2013 and (UE) 2015/1830 Regulation of the Commission 8 of July of 2015 [38, 39].

The content of free fatty acids is expressed as acidity calculated conventionally. For this determination 20 g of oil is dissolved in 50 mL of the previously neutralized mixture of ethanol-ether (1 : 1 v/v). The sample is titrated with potassium hydroxide using phenolphthalein as indicator. The acidity is expressed as percentage of oleic acid.

Peroxide value (PV) was expressed as milliequivalent of active oxygen per kilogram of oil. 1.2–2.0 g of oil was dissolved in a mixture of chloroform and acetic acid (2 : 3 v/v). Then 1 mL of KI saturated solution was added and 75 mL of deionized water was also added after stirring and 5 min in darkness. Finally the sample is titrated with sodium thiosulphate and starch solution as indicator.

*K*_232_ and *K*_268_ extinction coefficients were calculated by spectrophotometric examination in the ultraviolet. 100 mg of oil was dissolved in 25 mL of isooctane and the extinction of the solution is then determined at the specified wavelengths with reference to pure solvent. Specific extinctions are calculated from the spectrophotometer readings (Varian Cary 4000) using a quartz cell with a 1 cm optical path.

The testing panel of The Technological Center of the Olive Grove and Olive Oil (CITOLIVA) carried out the sensory characterization and linked the flavor stimuli of the EVOOs with a numeric scale according to the analytical methods described in the European Commission Regulation number 2568/91 and subsequent amendments (number 1348/2013) [39].

### 2.3. Purity Parameters

The gas chromatography (GC) is the analytical technique used to determine the qualitative and quantitative fatty acid methyl-esters composition [39]. The samples were prepared by shaking a solution of oil in heptane (0.1 g in 2 mL) with 0.2 mL of 2 N methanolic potassium hydroxide stirred during 30 seconds. The methyl-esters were analyzed from the supernatant solution by gases chromatographer Shimadzu GC-2014AF/SP. The oven temperature was set at 185°C, and the injector and detector temperatures were set at 220°C. The chromatograms were read in ascending order of number of carbons and unsaturations.

### 2.4. Minor Components

The tocopherols were evaluated by HPLC following the method described by Cunha et al. [40] on a Shimadzu HPLC mod. Prominence serie 20. A Sigma-Aldrich (250 mg/L) *α*-tocopherol in acetonitrile was frozen at −20°C until being used as internal pattern. 0.2 g of oil was dissolved in 10 mL of n-hexane and 20 *μ*L of sample was analyzed. As mobile phase hexane was used: isopropanol (97 : 3 v/v) in isocratic conditions and 1 mL/min of flow rate. The chromatograms were recorded at 275 nm. The results are expressed as mg of *α*-tocopherol per oil kg.

Chlorophyll and carotenoids compounds were determined at 472 and 670 nm (maximum absorption wavelength of pheophytin and lutein) in cyclohexane by the method of Mínguez [41], by a Varian Cary 4000 spectrophotometer. The chlorophyll and carotenoid concentration was expressed as milligrams of pigment per kilogram of oil.

The total polyphenols were determined following the method described by Vázquez-Roncero et al. [42], using the Folin-Ciocalteau reagent. The olive oil sample was dissolved in hexane and the phenolic compounds were extracted by triple extraction by methanol : water (60 : 40 v/v), by decantation. A mixture of 5 mL of polyphenols extract was mixed in 35 mL of water and 2.5 mL of Folin-Ciocalteau reagent. The mixture was homogenized by agitation and finally 5 mL of Na_2_CO_3_ saturated was added. The absorption of the solution was measured at 725 nm. Results were given as mg/kg of caffeic acid.

### 2.5. Statistical Analysis

The multifactorial analysis of variance (ANOVA) was performed using Statgraphics Centurion software. This analysis is designed to build a statistical model describing the impact of two or more categorical factors of a dependent variable. Tests were done to find out any significant differences between the media values and any interactions between the factors. The ANOVA table shows the decomposition of the variability of the measurable parameter in contributions of different factors. As the sum of the squares (type III) was selected, the interaction of each factor is determined eliminating the other factors' effects. The values of *P* indicate the statistical significance of each factor.

## 3. Results and Discussion

Three monovarietal extra virgin olive oils have been studied in this research, arbequina, picual (two brands A and B), and hojiblanca, making a total of four EVOOs. This section describes the results obtained and discusses the influence of the experimental conditions on the quality parameter and minor components of the EVOOs.

Tables 1 and 2 show the values of the quality parameters and minor components of each sample of extra virgin olive oil at the beginning of the assay (control sample) and after each treatment (T1 to T4).

### 3.1. Acidity

The acidity of the arbequina and hojiblanca EVOO control samples was lower than the fixed limit by the European and International Regulations, while, for both picual brands (A and B), the acidity values were slightly higher, 0.09 and 0.11, respectively.

As can be seen, the initial acidity grade of the arbequina and hojiblanca olive oils was unaltered after the heating treatment, and their values ranged from 0.08 to 0.09. According to this parameter these samples preserve the extra virgin olive oil category. However, the samples of picual variety changed their designation to “virgin” due to an increase of the AG of 0.13 and 0.14, respectively.

This trend has been also noted by other authors who studied the olive oil stability after heating with different exposure times in microwave and conventional oven [34, 43–46].

The results indicate that the treatment at 40°C produces the largest increase of the acidity with increases of 86%, while treatment at 60°C during two and three weeks causes an increase of 67% and 64%, respectively. The acidity of the arbequina variety olive oil is the least affected with the treatment, two of the samples are unaffected, and the rest shows an acidity increase of 15% in comparison to 54% of the picual variety brand B, 37% of picual variety brand A, and 204% of hojiblanca variety.

### 3.2. Peroxide Value (PV)

According to the current legislation, at the beginning of the storage the oil samples had low levels of PV and fall into the extra virgin olive oil category. These initial peroxide values were 10.60 picual A, 8.44 picual B, 5.19 hojiblanca, and 9.44 arbequina (Table 1).

A significant decrease in PV occurred in the picual and arbequina samples after the treatment with a decrease of 20–30%. However no significant changes were observed for the hojiblanca variety samples. These results confirm that all the olive oil varieties studied preserve their extra virgin olive oil category after the time-temperature treatments.

**Figure 1:**
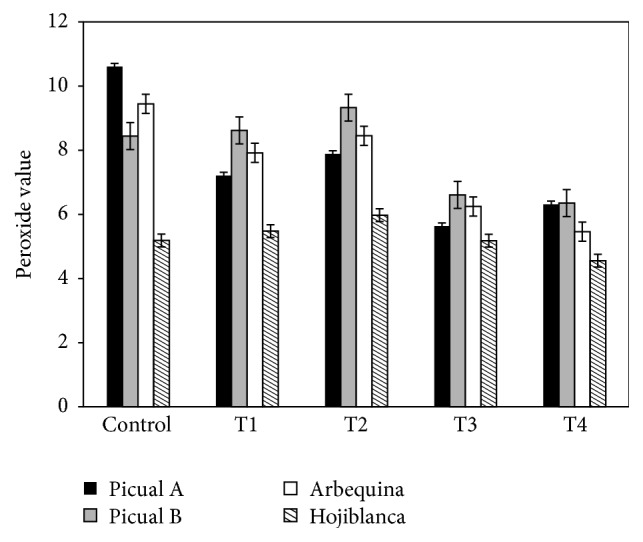
Influence of the heating treatments on the peroxide value of the olive oils.

Figure 1 shows that the treatment at 60°C causes the greatest impact in the PV decreasing the PV levels in all the olive oil samples studied.

Comparing the results with other authors who use different energy sources [37], no significant changes in PV with time are observed in microwave heating while PV decreases are detected after heating in conventional oven or frying (about 50%).

However, there is no consensus in literature about the PV evolution of olive oils after heating treatments. Albi et al. [34] reported a slight increase of the PV of the extra virgin olive oil after 120 min of microwave heating at 170°C while this parameter decreases in a virgin olive oil after being exposed to the same conditions. Cossignani et al. [31] also noted an increase of the PV of the extra virgin olive oil after a microwave heating of 8 minutes.

The behavior of the PV could be explained by the evolution of the oxidation process, the PV increase is caused by the hydroperoxide formation, and the appearance of secondary oxidation products reduces the PV.

### 3.3. *K*_232_ and *K*_268_ Extinction Coefficients

The UV spectrophotometric study provides information about the degree of olive oil oxidation and therefore its quality [38]. The *K*_232_ extinction coefficients indicate the conjugation of dienes formation of the polyunsaturated fatty acids (primary oxidation), while the *K*_268_ extinction coefficients are indicators of the presence of secondary oxidation products, including conjugated trienes and carbonyl compounds.

The maximum values permitted for *K*_232_ and *K*_268_ are, respectively, 2.50 and 0.22 for extra virgin olive oils and 2.60 and 0.25 for virgin olive oil, respectively.

**Figure 2:**
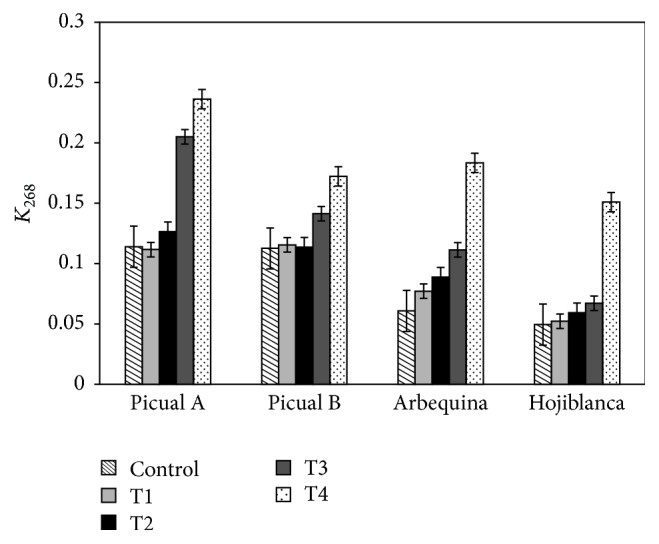
Variation of *K*_268_ with the treatments.

Table 1 and Figure 2 show the *K*_232_ and *K*_268_ extinction coefficient evolution with the heating treatment.

All the olive oil samples presented *K*_232_ and *K*_268_ values lower than the maximum limits established for the extra virgin category (2.50 and 0.22 for *K*_232_ and *K*_268_, resp.) at the initial stage and after each time-temperature treatment.

However only one sample of picual variety presents a *K*_268_ value of 0.24 after three weeks at 60°C, and this value is the nearest to the maximum limit stablished for EVOO category.

In relation to the effect of the heating treatment the highest increment of the coefficient *K*_232_ is observed after 3 weeks at 60°C for all the samples with the exception of the picual A olive oil whose coefficient reaches the highest increase after 2 weeks at 60°C. The treatments at 60°C have the greatest impact on the *K*_268_ coefficients (Figure 2).

The effect of the heating on the quality parameters of the olive oil has been also studied by several authors with similar results. Malheiro et al. [32] reported increases of the *K*_232_ coefficient after microwave heating. In this study all oils presented *K*_232_ higher than 3.1 after 15 min of heating that indicates an accelerated degradation process and therefore the olive oils no longer correspond to the extra virgin category.

This behavior is observed in all the olive oil varieties studied. No relevant changes were observed in the olive oils after the treatment in the conventional oven or during frying. However a significant increase of the *K*_268_ extinction coefficient is observed in all the varieties after each heating treatment in both the conventional oven heating and microwave heating. This increase with the time is due to the formation of oxidation compounds which explained the PV decrease [32, 37, 43].

### 3.4. Sensory Evaluation

The sensory evaluation of the olive oils was developed following the Regulation (EEC) number 2568/91 on the characteristics of olive oil and olive-residue oil and on the relevant methods of analysis and the amendment in the Regulation (EEC) number 1348/2013 [39] which graded the olive oils categories in line with the median of the defects and the median for “fruity.”

**Figure 3:**
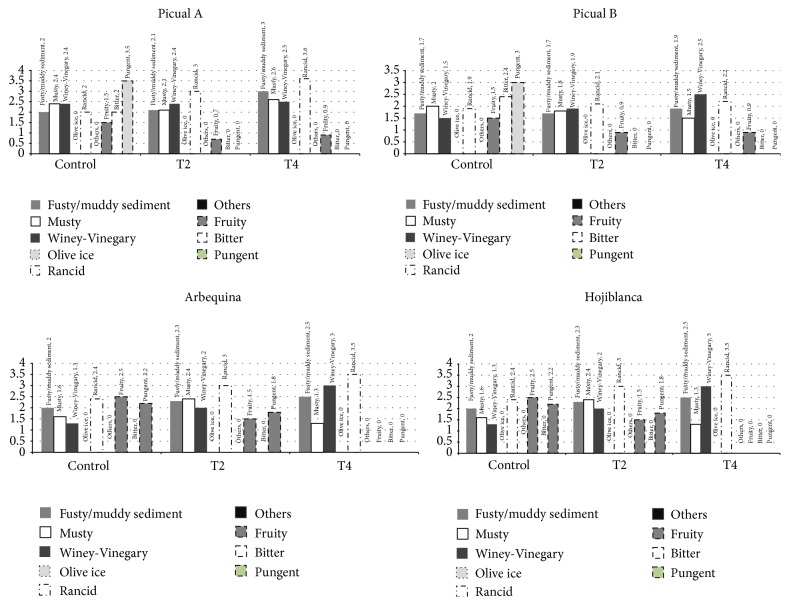
Influence of the heating treatments on the sensory analysis. Positive features and defects.

**Figure 4:**
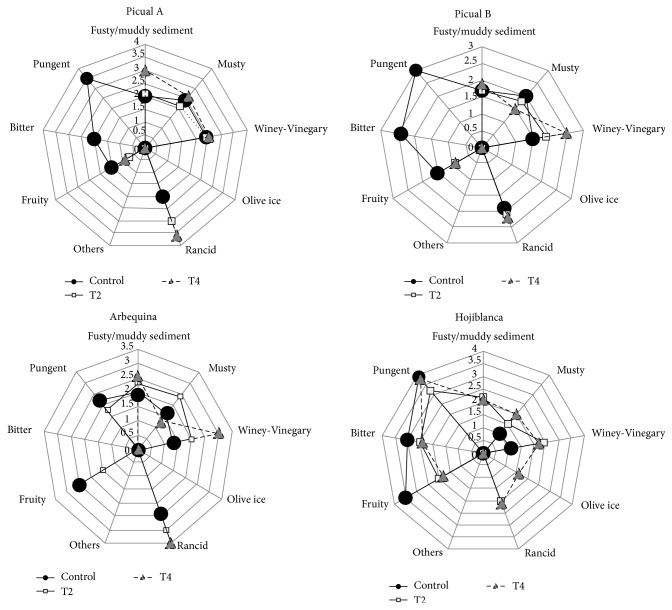
Sensory profile of the olive oils after 3 weeks at 40°C (T2) and 3 weeks at 60°C (T4).

Figure 3 shows the median of the positive features and defects selected for each stove heating treatment. It can be observed that none of the olive oils studied preserves the “extra virgin” category, some of them became virgin category, and the picual and arbequina varieties of olive oils became “lampante” category after three weeks at 60°C.  Figure 4 shows the sensory profiles of all the olive oils studied.

### 3.5. Fatty Acid Profile

The changes of the fatty acid percentages could be probably due to the chemical reactions (oxidation, hydrolysis) as a consequence of the heating treatments. The thermolability or volatility of these compounds has great influence [44].

As Table 2 shows, the fatty acid composition was unaltered for all the olive oil varieties. These profiles were in all the samples according to the “extra virgin” category in compliance with the requirements of the European Regulations [38] for the EVOO denomination. Only the arachidic acid and gadoleic acid content were initially slightly higher than the EU limits of 0.60 and 0.40, respectively. The results of this research are similar to other studies [35] where the fatty acid profiles of different vegetable oils after microwave and conventional heating treatments were unchanged. Table 2 shows medium values and the variability expressed as typical deviation.

### 3.6. Minority Compounds: Pigments, Polyphenols, and Tocopherols

**Figure 5:**
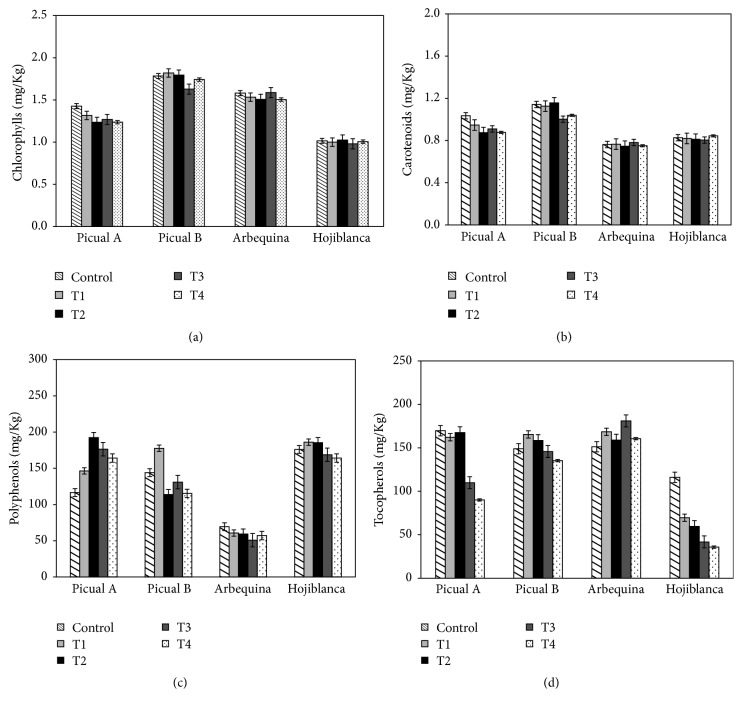
Influence of the heating treatments on the minority compounds: (a) chlorophylls, (b) carotenoids, (c) phenols, and (d) tocopherols.

Unlike the results obtained by Malheiro et al. [32] and Yahyaoui et al. [45], the heating treatments at 40°C and 60°C for two and three weeks have no influence on both chlorophyll and carotenoid concentrations (Figures 5(a) and 5(b)). Indeed they can be considered constant values for all the oils, except in the picual sample whose value diminished by 50% as time and temperature increase.

The polyphenols content expressed as mg of caffeic acid per kg of oil remains relatively unchanged with the heating treatments in stove. However, other researches [34, 36, 45, 46] have shown that the microwave heating treatments decrease the antioxidant components content between 55% and 85%.

With regard to the content of tocopherols, expressed as mg/kg of *α*-tocopherol, different trends are shown in Figure 5(d). The tocopherol content is unchanged for the picual B sample. A rise of this content is observed for the arbequina variety oils and picual B and hojiblanca variety oils decrease this content between 47% and 70%, respectively, as time and temperature increase, with a maximum value after 3 weeks at 60°C.

### 3.7. Influence of the Variety, Time, and Temperature

The results of the multifactorial statistical analysis, ANOVA, with the objective of study being the influence of the three variables studied (variety, time, and temperature) on the quality parameters are shown in Table 3. The *P* values show that the most significant variable is the variety with *P* < 0.05 for all the parameters. Temperature shows *P* < 0.05 for 50% of the parameters and time is the factor that has less significance. The parameters experiencing high variability are *K*_232_, *K*_268_, peroxide, and tocopherols, followed by acidity grade, polyphenols, and carotenoids. The olive variety is the factor with the major influence on the variability.

## 4. Conclusions

This research simulates extreme storage, transport, or conservation conditions with high temperatures similar to the temperatures reached in countries with hot climate. The effects of these conditions on the quality of three olive oils varieties are conclusive about the quality of the olive oils of the three varieties studied and they can be extrapolated to other varieties for similar conditions.

The picual variety, in terms of quality parameters, shows changed values of AG, PV, *K*_232_, and *K*_268_ compared to the limits established by the European Regulations for EVOO. The arbequina variety has similar behavior but only for the PV and the hojiblanca variety maintains its parameters within the EVOO category. However the sensory analysis of all the varieties samples determines that the oils became “virgin” and even “lampante” category after the heating treatments. The fatty acid profile is practically unchanged for the three varieties.

In relation to the minority compounds, the pigments are unaltered with the exception of picual A. The polyphenols content is also unaffected by the heating treatments. However the tocopherols content changes considerably with the exception of the picual B.

In view of the results the sensory analysis and the minority compounds are the most influenced after the treatments and due to their great influence in the preservation of the EVOO category the interest of this study to control all the traceability steps of the olive oil for commercialization is remarkable. The variance analysis (ANOVA) indicates that all the factors studied (olive variety, time, and temperature) have a great influence but the olive variety is the most relevant. It should be mentioned that the quality parameters more affected (*K*_232_, *K*_268_, and peroxide) show the oxidation state of the olive oil.

## Figures and Tables

**Table 1:** Quality parameters.

	Control sample	T1	T2	T3	T4
*Picual A*					
Acidity grade	0.090 ± 0.001	0.140 ± 0.001	0.180 ± 0.002	0.130 ± 0.001	0.130 ± 0.001
*K*_232_	1.77 ± 0.05	1.66 ±0.01	1.74 ± 0.01	1.84 ± 0.02	1.71 ± 0.01
*Picual B*					
Acidity grade	0.110 ± 0.001	0.140 ± 0.002	0.110 ± 0.001	0.110 ± 0.001	0.110 ± 0.002
*K*_232_	1.89 ± 0.04	1.79 ± 0.06	1.80 ± 0.02	1.88 ± 0.07	1.97 ± 0.06
*Arbequina*					
Acidity grade	0.060 ± 0.003	0.080 ± 0.001	0.090 ± 0.001	0.080 ± 0.002	0.090 ± 0.003
*K*_232_	0.93 ± 0.01	0.96 ± 0.04	0.98 ± 0.01	0.94 ± 0.01	2.15 ± 0.02
*Hojiblanca*					
Acidity grade	0.030 ± 0.002	0.100 ± 0.004	0.090 ± 0.003	0,080 ± 0.002	0.080 ± 0.002
*K*_232_	0.72 ± 0.03	0.73 ± 0.02	0.78 ± 0.02	0.72 ± 0.03	1.84 ± 0.05

Results expressed as mean value ± typical deviation.

**Table 2:** Fatty acid composition.

	Control sample	T1	T2	T3	T4
*Picual A*					
C16:0	10.52 ± 0.12	10.41 ± 0.04	10.38 ± 0.05	10.36 ± 0.35	10.43 ± 0.41
C16:1	0.81 ± 0.01	0.81 ± 0.01	0.82 ± 0.01	0.81 ± 0.03	0.80 ± 0.01
C17:0	0.050 ± 0.007	0.050 ± 0.003	0.050 ± 0.004	0.050 ± 0.006	0.050 ± 0.006
C17:1	0.090 ± 0.001	0.090 ± 0.003	0.100 ± 0.001	0.100 ± 0.001	0.090 ± 0.001
C18:0	3.40 ± 0.03	3.30 ± 0.05	3.24 ± 0.02	3.23 ± 0.04	3.33 ± 0.02
C18:1	80.47 ± 0.45	80.01 ± 0.08	80.05 ± 0.09	80.20 ± 0.17	79.98 ± 0.08
C18:2	4.06 ± 0.03	4.08 ± 0.03	4.04 ± 0.01	4.04 ± 0.03	4.02 ± 0.01
C20:0	0.620 ± 0.023	0.640 ± 0.003	0.660 ± 0.002	0.650 ± 0.005	0.640 ± 0.001
C20:1	0.41 ± 0.01	0.41 ± 0.01	0.42 ± 0.05	0.40 ± 0.01	0.40 ± 0.04
C18:3	0.23 ± 0.03	0.24 ± 0.01	0.28 ± 0.04	0.26 ± 0.01	0.27 ± 0.03
*Picual B*					
C16:0	10.92 ± 0.05	10.73 ± 0.09	10.41 ± 0.22	10.60 ± 0.16	6.01 ± 0.02
C16:1	0.90 ± 0.01	0.89 ± 0.07	0.91 ± 0.02	0.90 ± 0.02	0.47 ± 0.42
C17:0	0.060 ± 0.005	0.040 ± 0.003	0.040 ± 0.001	0.050 ± 0.001	0.040 ± 0.007
C17:1	0.090 ± 0.003	0.130 ± 0.039	0.090 ± 0.004	0.080 ± 0.001	0.090 ± 0.004
C18:0	3.11 ± 0.02	3.08 ± 0.04	2.90 ± 0.04	3.03 ± 0.06	3.33 ± 0.21
C18:1	80.14 ± 0.080	80.39 ± 0.082	80.89 ± 0.232	80.57 ± 0.230	85.09 ± 4.956
C18:2	3.44 ± 0.09	3.43 ± 0.02	3.47 ± 0.03	3.44 ± 0.06	3.60 ± 0.20
C20:0	0.660 ± 0.007	0.660 ± 0.003	0.670 ± 0.011	0.670 ± 0.003	0.70 ± 0.041
C20:1	0.410 ± 0.001	0.400 ± 0.002	0.380 ± 0.004	0.410 ± 0.008	0.420 ± 0.028
C18:3	0.280 ± 0.018	0.260 ± 0.005	0.250 ± 0.001	0.270 ± 0.001	0.270 ± 0.015
*Arbequina*					
C16:0	11.45 ± 0.02	11.06 ± 0.24	10.80 ± 0.51	11.31 ± 0.05	12.06 ± 0.07
C16:1	1.38 ± 0.07	1.44 ± 0.07	1.42 ± 0.02	1.43 ± 0.07	1.40 ± 0.01
C17:0	0.130 ± 0.010	0.120 ± 0.003	0.130 ± 0.001	0.120 ± 0.009	0.130 ± 0.001
C17:1	0.280 ± 0.009	0.260 ± 0.008	0.270 ± 0.001	0.270 ± 0.001	0.270 ± 0.002
C18:0	1.95 ± 0.04	2.03 ± 0.06	2.03 ± 0.01	2.06 ± 0.07	2.07 ± 0.04
C18:1	74.13 ± 0.02	74.43 ± 0.17	74.64 ± 0.34	74.18 ± 0.04	73.62 ± 0.06
C18:2	9.02 ± 0.07	9.10 ± 0.04	9.12 ± 0.10	9.00 ± 0.04	8.88 ± 0.07
C20:0	0.690 ± 0.014	0.650 ± 0.005	0.640 ± 0.002	0.670 ± 0.032	0.630 ± 0.002
C20:1	0.410 ± 0.015	0.410 ± 0.002	0.430 ± 0.020	0.440 ± 0.018	0.420 ± 0.001
C18:3	0.350 ± 0.020	0.370 ± 0.020	0.370 ± 0.010	0.360 ± 0.016	0.360 ± 0.002
*Hojiblanca*					
C16:0	9.93 ± 0.02	8.72 ± 0.01	10.12 ± 0.06	10.47 ± 0.08	10.44 ± 0.04
C16:1	0.820 ± 0.001	0.850 ± 0.008	0.850 ± 0.007	0.820 ± 0.006	0.820 ± 0.006
C17:0	0.100 ± 0.002	0.100 ± 0.001	0.100 ± 0.002	0.110 ± 0.002	0.450 ± 0.002
C17:1	0.570 ± 0.003	0.180 ± 0.003	0.170 ± 0.001	0.170 ± 0.001	0.240 ± 0.004
C18:0	3.18 ± 0.05	3.03 ± 0.01	3.08 ± 0.02	3.32 ± 0.01	3.31 ± 0.02
C18:1	77.25 ± 0.07	78.82 ± 0.06	77.63 ± 0.03	77.14 ± 0.07	76.82 ± 0.32
C18:2	6.58 ± 0.04	6.78 ± 0.01	6.56 ± 0.05	6.48 ± 0.02	6.45 ± 0.02
C20:0	0.760 ± 0.005	0.780 ± 0.003	0.760 ± 0.001	0.750 ± 0.003	0.730 ± 0.001
C20:1	0.430 ± 0.003	0.420 ± 0.004	0.420 ± 0.001	0.440 ± 0.001	0.430 ± 0.006
C18:3	0.300 ± 0.003	0.310 ± 0.008	0.300 ± 0.007	0.300 ± 0.000	0.310 ± 0.008

**Table 3:** Analysis of variance (ANOVA).

Parameters	AG	PV	*K*_232_	*K*_268_	Polyphenols	Chlorophylls	Carotenoids	Tocopherols
	*P*	*P*	*P*	*P*	*P*	*P*	*P*	*P*
Main effects								
Variety	0.0000	0.0000	0.0000	0.0000	0.0000	0.0000	0.0000	0.0000
Temperature	0.0000	0.0000	0.0000	0.0000	0.0422	0.0696	0.0252	0.0000
Time	0.2936	0.0135	0.0000	0.0000	0.3150	0.5975	0.6030	0.0000
Interactions								
Variety-temperature	0.0011	0.0001	0.0027	0.0000	0.4174	0.0542	0.0025	0.0000
Variety-time	0.0000	0.1596	0.0001	0.0000	0.0078	0.1279	0.0909	0.4787
Temperature-time	0.0956	0.0071	0.0000	0.0000	0.8857	0.3909	0.4272	0.0272

The *P* values show the statistical significance of each factor. Interaction level 2: nivel de integración “2.”

For *P* < 0.005 the effects have a statistical significance over each parameter with a statistical confidence of 95.0%.
